# DEXAMETHASONE: CHONDROPROTECTIVE CORTICOSTEROID OR CATABOLIC KILLER?

**DOI:** 10.22203/eCM.v038a17

**Published:** 2019-11-22

**Authors:** R. Black, A. J. Grodzinsky

**Affiliations:** 1 Department of Biological Engineering, Massachusetts Institute of Technology, Cambridge, MA, USA; 2 Department of Mechanical Engineering, Massachusetts Institute of Technology, Cambridge, MA, USA; 3 Department of Electrical Engineering and Computer Science, Massachusetts Institute of Technology, Cambridge, MA, USA

**Keywords:** Cartilage-general, cells/tissues-injury, cells/tissues-cartilage, ECM-general, ECM-proteoglycans, cells/tissues-proliferation, cells/tissues chondrocytes

## Abstract

While glucocorticoids have been used for over 50 years to treat rheumatoid and osteoarthritis pain, the prescription of glucocorticoids remains controversial because of potentially harmful side effects at the molecular, cellular and tissue levels. One member of the glucocorticoid family, dexamethasone (DEX) has recently been demonstrated to rescue cartilage matrix loss and chondrocyte viability in animal studies and cartilage explant models of tissue injury and post-traumatic osteoarthritis, suggesting the possibility of DEX as a disease-modifying drug if used appropriately. However, the literature on the effects of DEX on cartilage reveals conflicting results on the drug’s safety, depending on the dose and duration of DEX exposure as well as the model system used. Overall, DEX has been shown to protect against arthritis-related changes in cartilage structure and function, including matrix loss, inflammation and cartilage viability. These beneficial effects are not always observed in model systems using initially healthy cartilage or isolated chondrocytes, where many studies have reported significant increases in chondrocyte apoptosis. It is crucially important to understand under what conditions DEX may be beneficial or harmful to cartilage and other joint tissues and to determine potential for safe use of this glucocorticoid in the clinic as a disease-modifying drug.

## Introduction

GCs, a family of steroid hormones, have been used since the 1950s for treating pain and inflammation in both RA and OA, diseases associated with cartilage degeneration and joint inflammation (Creamer, 1999; Hollander *et al.*, 1951). In the early twentieth century, new treatments for RA using extracts of animal adrenal cortical tissue led to the need for larger quantities of synthetic steroids. By the 1960s, rapid advances in chemical synthesis of GCs resulted first in synthetic cortisone, then hydrocortisone, fluorohydrocortisone, prednisone, prednisolone, triamcinolone, methylprednisolone and, finally, DEX, the latter acknowledged to be the most potent member of the GC family (Benedek, 2011).

Although the beneficial effects of GCs are now cited as well-known and accepted, there remains much controversy in the field about their prescription for patients (Hollander, 1960; Zeng *et al.*, 2019). Harmful side effects from systemic delivery of GCs were recognised early on, leading to the pioneering development of i.a. injection for steroid therapy in the 1950s (Jüni *et al.*, 2015). However, even with i.a. delivery of GCs, their mechanism of action in both diseased and healthy joints is not well understood. GCs can affect a variety of cellular pathways, suppressing inflammation with sometimes unknown off-target effects (Saklatvala, 2002). Even among the most recent clinical trials, there are conflicting results over their safety and efficacy, often due to low-quality evidence on their effects or different methodologies used (Conaghan *et al.*, 2018).

The greatest concern over GC use in the context of arthritis treatment is their potential for catabolic effects on cartilage. While several clinical studies have shown significant short-term reduction in pain after treatment with GCs (Arroll and Goodyear-Smith, 2004; Conaghan *et al.*, 2018; Hepper *et al.*, 2009), others have revealed that with repeated i.a. injections at typically high clinical doses, GCs can lose their analgesic effects (McAlindon *et al.*, 2017; Wernecke *et al.*, 2015). In addition, a recent report has suggested that repeated i.a. injections every 3 months over a 2-year period cause macroscopic changes in cartilage, *e.g.* a loss of knee cartilage volume and thickness as measured by MRI (McAlindon *et al.*, 2017). Animal and *in vitro* studies have also raised critically important issues regarding dose and duration of repeated injections. The often-cited study of Mankin *et al.* (1972) reported a significant loss of cartilage sGAG and suppressed matrix biosynthesis following daily intramuscular injections of 4.5 mg/kg cortisone into rabbits over a 9-week study duration. In contrast, Gibson *et al.* (1977) injected prednisolone (3.5 mg/kg) into mature monkey knees only once, twice or six times over a 12-week period and reported essentially no changes in injected knees as compared to controls. Was the difference in cartilage response associated with the animal species, dose, number of injections or choice of GC? Even after so many decades of clinical use of GCs, questions remain as to what mechanisms GCs act on in cartilage and surrounding joint tissues and under what dosing regimens GCs remain safe for patient use (Arroll and Goodyear-Smith, 2004; Wernecke *et al.*, 2015).

While the most commonly used GCs today include prednisolone, triamcinolone, betamethasone and DEX, they are reported to have differential effectiveness at different doses from one another, further complicating the ability to compare and interpret treatment protocols and mechanisms of action. Therefore, the present review focuses on the effects of only one member of the GC family, DEX. This narrower focus enabled a comparison of studies on the effects of a single GC across different biological systems, from isolated cells to intact organ culture explants, animal studies *in vivo* and human clinical trials.

The controversy over DEX begins at the level of clinical trials. Two recent trials studying changes in pain post-knee arthroplasty surgery have used the same DEX dose, but only one found a significant reduction in pain after DEX administration (Web ref. 1; Web ref. 2). Unlike other GCs, no clinical trial has been completed that assesses the effects of DEX on cartilage structure or function; however, several recent studies have shown that DEX may have chondroprotective effects on cartilage in the context of PTOA when using *in vitro* human cartilage explant models [Li *et al.*, 2015; Lu *et al.*, 2011; Wang *et al.* (2014) Dexamethasone treatment alters the response of human cartilage explants to inflammatory cytokines and mechanical injury as revealed by discovery proteomics. Osteoarthritis Cartilage 25: S381–S382]. These results present the possibility of repurposing and using DEX as a DMOAD in contexts such as PTOA, where the anti-inflammatory and chondroprotective effects of DEX may prevent the progression of the disease. However, more studies must be performed to clarify the timing and dosage appropriate to obtain these effects *in vivo*.

While these possibilities are exciting, caution must be taken until possible chondrotoxic effects of DEX are better understood. Several studies using healthy human chondrocytes have reported that even low doses of DEX cause cell death and reduce cell proliferation, suggesting potential cytotoxic and catabolic side effects. However, the observed effects of DEX depends greatly on dose, model, duration of treatment and context (*e.g.* isolated cells *versus* intact cartilage). Thus, study conclusions often differ greatly, complicating the discussion on the safety and efficacy of this drug. It is particularly important to understand appropriate use in human disease due to the variety of reported side effects of GC treatment, including hypertension, adrenal gland depression, psychological disturbances, Cushing’s syndrome, osteoporosis and susceptibility to infections as a result of immunosuppression (Bordag *et al.*, 2015; Manson *et al.*, 2009).

The present narrative review presents literature on DEX effects in both arthritic and healthy model systems. The potential disease-modifying and catabolic effects of DEX are described and the need for dose-duration studies in humans are emphasised to ascertain under what conditions DEX may be safe to use and whether it may have uses as a DMOAD in treating OA beyond analgesia and reducing inflammation. A search for “dexamethasone effects on cartilage” in PubMed and Cochrane Library databases returned 451 results, 46 of which were selected for their relevance to the scope of the review. Criteria for inclusion were: (1) use of DEX alone (*i.e.* not in combination with another drug or with a method of delivery that could affect the distinction between the effects of DEX or the effects of a carrier) and (2) use of either an animal model (*i.e.* measuring changes to cartilage and OA scores), cartilage explants or chondrocytes, whether primary or derived from a chondrogenic line.

### DEX and animal models of arthritis

Studies in animals (Table 1) have suggested a promising role for DEX as a potent preventative measure against arthritis progression but, conversely, suggest that it may damage healthy cartilage at certain doses and durations. In a collagen-induced mouse model of RA, Rauchhaus *et al.* (2009) found that daily intravenous injections of free 1.6 mg/kg DEX or a single injection of 0.4–4 mg/kg liposomalencapsulated DEX both reduce the frequency of arthritis occurrence and lower its severity. However, the persistent anti-inflammatory effects gained by single-dose liposomal encapsulation hinted at the importance of drug delivery. Islander *et al.* (2011) used a mouse postmenopausal OA model and found that daily 125 μg intraperitoneal injections of DEX protect against arthritis and joint destruction. A rat meniscal transection OA model showed that daily oral gavage treatments with 0.1 mg/kg DEX starting 11 d after surgery decrease animal pain response in the affected paw, lowers inflammation and macrophage infiltration and partially rescues PG loss in the joint cartilage (Ashraf *et al.*, 2011). Another similar rat study using an AA model treated with 0.15 mg/kg daily DEX through oral gavage starting 13 d after AA induction found less swelling in the paws of rats treated with DEX. The same study showed that there is also a significant overlap in genes downstream of inflammatory mediators regulated by DEX and the network of genes affected by the development of AA but did not explore the identity of these genes or how they may provide protection to cartilage (Wang *et al.*, 2017a). Rabbits injected i.a. with 0.5 mg/kg DEX once before and every 3 d after a surgery-induced PTOA showed protection of articular cartilage at 3 weeks after surgery, with Mankin scores equal to the control rabbits (Huebner *et al.*, 2014). Heard *et al.* (2015) used the same rabbit surgical model, with a single i.a. DEX injection at the time of surgery (0.5 mg/kg), and showed significant improvement of histological grading of cartilage and synovium 9 weeks post-surgery. This is due to improvement in safranin-O staining of GAGs, while DEX treatment does not influence the medial structure grade. Malfait *et al.* (2009) credited such a protective effect of DEX on inhibition of GAG loss to protection against aggrecan proteolysis in the cartilage matrix, as demonstrated in their rat model of OA using i.a. injections of TNFα followed by 16 h of intravenous infusion with 0.0011 mg/mL DEX in saline.

The most in-depth analysis of cartilage structure after DEX treatment in an animal model was performed by Jaffré *et al.* (2003), who used quantitative ultrasound measurements and picrosirius red staining to study the surface and internal structure of cartilage after inducing synovitis and significant loss of cartilage sGAG in rats with injections of zymosan. Daily i.a. injections of 0.1 mg/kg DEX prevent knee swelling and histological changes such as loss of hypertrophic cells and surface alterations, seen by toluidine blue staining, and maintain PG content at control levels. Using ultrasound measurements, they demonstrated that DEX returns the integrated reflection coefficient of the OA-challenged cartilage to control levels after 14 d, suggesting that DEX restores the microarchitecture and smoothness of the superficial layer after zymosan challenge. However, after 2 weeks of DEX treatment, the internal collagen network of the cartilage is markedly changed, with thinner and more disperse collagen fibres, a greater change than the effect of arthritis induction alone.

While DEX appears to provide protection against cartilage degradation, the results of Jaffré *et al.* suggest that there may be some dysregulation of the internal structure of cartilage organisation. It is also important to note the degenerative side effects of long-term daily DEX treatment: extended high doses of DEX can retard growth, which was verified by Jaffré *et al.* (2003) with significantly lower knee sizes and weights in DEX-treated rats. The rabbits in the study by Huebner *et al.* (2014) had extensive damage to their organs after DEX treatment: lungs, livers and kidneys appeared fibrotic and had large necrotic areas, suggesting serious systemic side effects to drug treatment. Rauchhaus *et al.* (2009) reported other systemic side effects in their mouse model, with reductions in body weight and lymphocyte count, neutrophilia and transient reduction in serum corticosterone levels. Two injections of 0.12 or 0.24 mg DEX reduce the growth of rat foetuses (an unsurprising finding given the well-established effect of GCs stunting growth in developing children) and cause the cartilage within the foetuses to have lower collagen content and reduced chondrocyte biosynthetic activity (Dearden *et al.*, 1986; Mushtaq and Ahmed, 2002). Taken together, these findings suggest the need for new approaches to deliver sustained low-doses of GCs intra-cartilage through single injection, thereby minimising unwanted exposure of other tissues (see Discussion; Krishnan *et al.*, 2018).

### DEX and healthy, non-arthritic animal models

Results from animal models have demonstrated that there is clearly a need to better understand the appropriate dosage and duration of treatment to prevent local and systemic side effects, especially when considering how the potential detrimental effects of DEX can extend to healthy cartilage. In models of OA, DEX can maintain the PG content of cartilage, but in healthy animals, DEX has been shown to damage cartilage and chondrocytes depending on dose and frequency of treatment. In a long-term study by Glade *et al.* (1983), daily intra-muscular injections of 0.5–5.0 mg/100 kg DEX into 6-month-old pony weanlings for up to 11 months caused massive degeneration of the still-developing injected joints, with articular lesions, fibrous scars and large necrotic areas in both cartilage and bone. These results were attributed to a depression of cartilage metabolism during the first 8 months of treatment, measured by ^35^S-methionine incorporation and LDH activity. In a small animal model but with a much higher DEX-dose, rats treated with 3.33 mg/kg DEX by systemic intramuscular injection for 5 weeks (1 mg/ week) showed reduced size of the rough endoplasmic reticulum and Golgi complex within articular chondrocytes, hypothesised to cause a reduction in protein synthesis (Podbielski and Raiss, 1985). DEX treatment resulted in an increased number of dead cells, attributed to its effect on lysosomal function as well as disrupting cell metabolism through reducing mitochondrial size. Annefeld and Erne (1987) also found a suppression of cartilage metabolism with a similar dose of intramuscular DEX (3 mg/kg), as well as higher rates of cell death within the cartilage after only 3 weeks. While it is difficult to extrapolate the doses used in animal models to what is appropriate for human patients, it is apparent that in some ways the effects of DEX may be chondroprotective under arthritic stresses but lead to catabolic degeneration and loss of viability in healthy tissue as dose and duration increase.

**Fig. 1.**
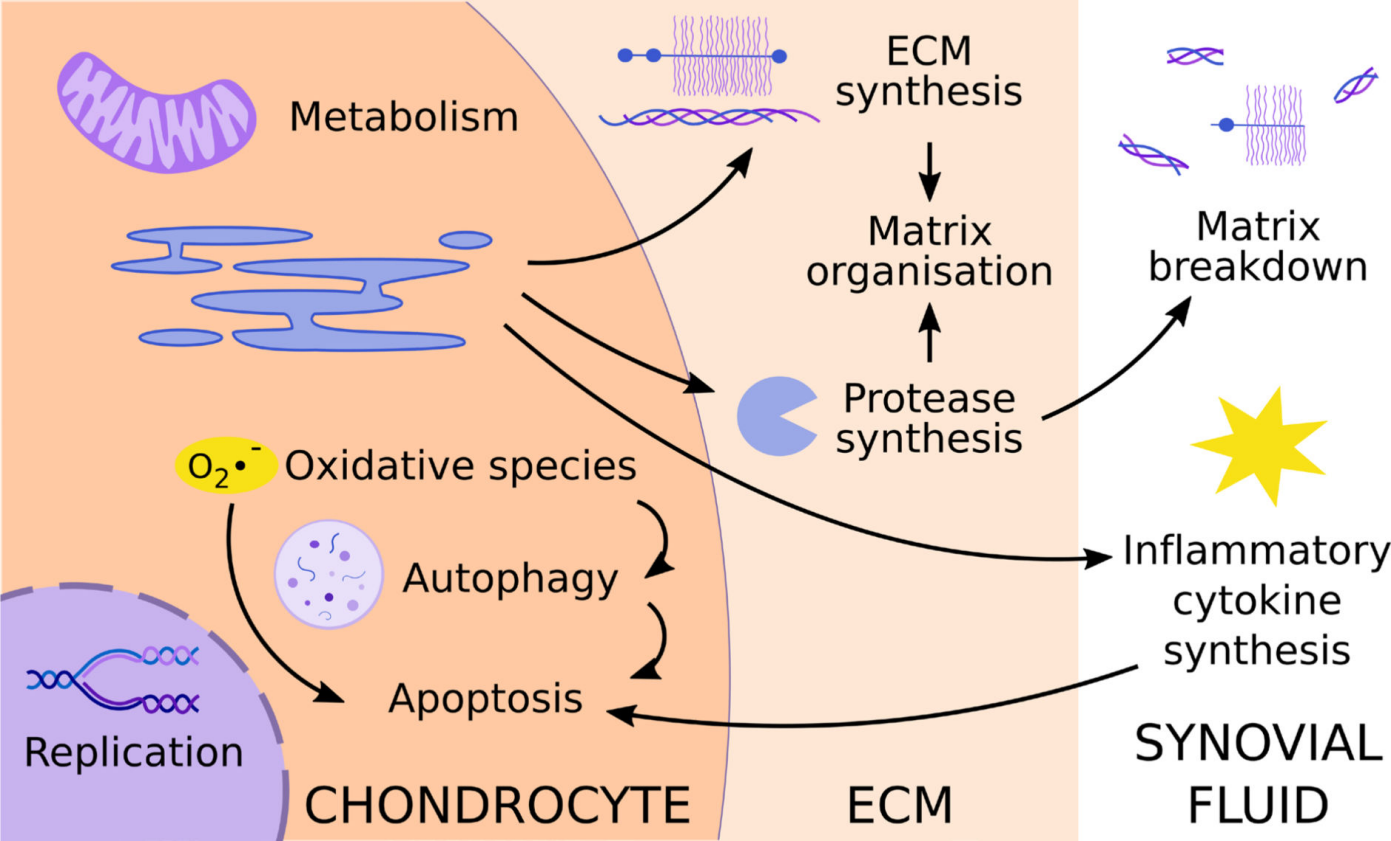
Biological processes identified as being affected by DEX either in healthy or diseased cartilage in studies using i*n vivo* models, cartilage explants or chondrocyte monoculture. DEX affects matrix organisation at the level of both ECM and protease synthesis, although studies often disagree on the specific up- or down- regulation of ECM specific components. There is a consensus that, under arthritic stresses, DEX prevents the upregulation of protease synthesis, which can prevent matrix loss. However, at higher doses, in healthy cartilage, DEX may increase the rate of matrix degradation or the organisation of the matrix itself. This could be due to effects on matrix components and proteases or due to intracellular effects on metabolism and the production of ROS that activate autophagy and lead to significant cell death. Data from *in vitro* studies have suggested that DEX maintains cell viability under arthritic stress, which could be linked to a DEX-induced reduction in inflammatory cytokine synthesis. Alternatively, the metabolic processes that DEX dysregulates in healthy tissue could serve to rescue changes in those processes after the initiation of arthritis. While the induction of autophagy in healthy tissue could lead to chondrocyte death and subsequent matrix breakdown, autophagy is suppressed in arthritic contexts, so DEX could serve to rescue these cellular processes in a diseased state. It remains to be seen whether DEX inhibits proliferation under arthritic stress and what role this might play in disease progression and whether the phenomenon of DEX-induced reduction of proliferation in healthy cartilage is due to cells becoming quiescent or senescent. Each possibility would have a significantly different biological outcome on cartilage exposed to DEX for an extended time.

Using animal models, chondrocyte death and matrix degradation are two of the most commonly reported outcome measures for determining the beneficial or harmful effects of DEX. However, there are many processes affecting these readouts that these studies do not always capture: DEX regulates many intracellular processes affecting cell viability and regulates both production of ECM proteins as well as proteases responsible for organising and breaking down the matrix. Some of the most well-studied processes are summarised in Fig. 1. The complex nature of the interactions between these effects complicates the interpretation of DEX effect on cartilage tissue and necessitates using *in vitro* cartilage explant and chondrocyte studies to interrogate the mechanistic effects of DEX.

### Cartilage explant models of arthritis

While there are no clinical data on the specific effects of DEX on cartilage in human patients, several studies have been performed on cartilage explants *in vitro* using tissue from human donors (Table 2). Li *et al.* (2015) demonstrated in an IL-1α-challenge of fullthickness near-normal human cartilage disks that culture with 100 nM DEX continuously over a 17 d treatment rescues GAG loss and maintains more viable cells within the cartilage, relevant to potential PTOA prevention. DEX also rescues the cytokine-induced decrease in sGAG synthesis, although not up to control levels. These results showed the beneficial effects of DEX to cartilage metabolism and ECM synthesis in a diseased state, the opposite of what is seen following long-term DEX treatment of healthy developing animal models (Glade *et al.*, 1983). Using human tissue in an 8 d TNFα + IL-6 challenge of normal human knee explants (± a single compressive impact injury relevant to PTOA), Lu *et al.* (2011) also found that continuous DEX treatment prevents GAG loss. Interestingly, in the same TNFα/IL-6 model, DEX greatly decreases the secreted levels of MMP-1 and −13, which correlates with decreases in fragments of ECM components released into the media, such as aggrecan, cartilage oligomeric matrix protein and collagen III neoepitopes [Wang *et al.* (2014) Dexamethasone treatment alters the response of human cartilage explants to inflammatory cytokines and mechanical injury as revealed by discovery proteomics. Osteoarthritis Cartilage 25: S381–S382].

Animal cartilage explant models corroborate some of the findings of human tissue and offer some additional mechanistic understanding of how the effects of DEX on arthritis progression are propagated at the transcriptional level. DEX returns GAG loss and sulphate incorporation to control levels in TNFαtreated bovine cartilage at DEX concentrations as low as 1 nM (Lu *et al.*, 2011). The reduction in GAG loss was suggested to be due to a suppressive effect of DEX on the activity of remodelling proteases, such as aggrecanases, through routes not limited to transcription alone, particularly in the case of ADAMTS-4 and −5, where mRNA transcripts remained elevated even after DEX exposure. A similar finding was reported by Busschers *et al.* (2010) using equine cartilage, where the addition of 100 nM or 1 μM DEX does not reduce the increase in ADAMTS-5 transcription after IL-1β challenge and levels of ADAMTS-4 mRNA transcripts are still elevated as compared to control, although decreased as compared to IL-1β alone. The same study reported no change in GAG loss into the medium or GAG content of articular and nasal cartilage explants after 72 h of exposure to DEX, differently from the findings of Lu *et al.* (2011) with bovine cartilage and from studies using human cartilage. Whether this is due to a difference in species, inflammatory cytokines used to model arthritis or culture length remains to be answered.

Li *et al.* (2015), in a 24 d IL-1α-challenge of bovine cartilage explants, observed that DEX maintains cell viability as measured by fluorescein diacetate and propidium iodide staining, consistent with a previous report of rescue of cell viability following mechanical impact injury using 100 μM DEX (D’Lima *et al.*, 2001). PCR results showed that after 4 d of IL-1α treatment, DEX greatly decreases the transcription of IL-6, ADAMTS-4, ADAMTS-5, MMP-3 and MMP-13 as compared to IL-1α alone without DEX, while also rescuing some expression of aggrecan and collagen II, although not up to control levels (Li *et al.*, 2015). It is of note that these results contradict the findings of Lu *et al.* (2011) on the effect of DEX on ADAMTS-4 and −5 transcription, although this discrepancy may be due to the different inflammatory cytokines used to stimulate cartilage breakdown and subsequent transcriptional pathways being activated. In another experiment with IL-1α and plasminogen challenge on rabbit cartilage, Saito *et al.* (1999) also found a decrease in the release of MMP-1 and −3 into the culture media at DEX concentrations as low as 1 nM. In addition, they showed some rescue of hydroxyproline release, a marker of collagen degradation.

Taken together, these results support the hypothesis that DEX helps to prevent the degradation of ECM components by inhibiting the increase in transcription or activity of matrix-degrading factors brought on during the progression of arthritis and by restoring some level of expression of the matrix components themselves (Fig. 1). However, there are still some discrepancies among the results of these experiments. While Li *et al.* (2015) reported a decrease in IL-6 expression in IL-1α-challenged bovine explants, Lu *et al.* (2011) did not see a change in IL-6 transcription with the addition of DEX to TNFα-challenged tissue. Garvican *et al.* (2010), using equine cartilage explants subjected to treatment with IL-1 and APC, observed that 1 μM DEX rescues hydroxyproline loss and decreases MMP-1, −3 and −13 transcription. However, DEX + APC treatment increases GAG release above the levels of IL-1/APC alone. This discrepancy may be due to the combined treatment with a protease as opposed to cytokines alone, as in the other models. Alternatively, GAG loss may be overestimated due to the analysis being based on GAG loss per wet weight of cartilage, a calculation that yields an exponentially increasing result for a linearly increasing loss of GAG.

### Non-arthritic cartilage explant models

Experiments treating healthy cartilage with DEX in the absence of an arthritic context have shown disagreements in the effects of DEX on ECM composition and tissue metabolism (Table 3). Two separate experiments with normal juvenile bovine explants treated with 100 nM DEX and one with equine cartilage explants treated with 100 nM or 1 μM DEX showed no change in the concentration of GAG per wet weight of the explants. The two bovine experiments reported no change in the release of GAGs into the culture medium (Bian *et al.*, 2010; Lu *et al.*, 2011), while equine cartilage explants showed a slight increase in GAG loss and a corresponding increase in the protease ADAMTS-5 transcript levels (Busschers *et al.*, 2010). However, Siengdee *et al.* (2015) found that a dose of 1.27 mM DEX, four orders of magnitude larger than that used by the three previously mentioned studies, causes porcine cartilage explants to release less GAG content into the culture medium and to maintain higher levels of safranin-O staining, indicative of larger concentrations of GAGs in the cartilage matrix.

The effect of DEX on the structure of the cartilage matrix may be dependent on the developmental state of the tissue, as Bian *et al.* (2010) observed an increase in equilibrium modulus and dynamic modulus of DEX-treated juvenile bovine cartilage, but not in adult bovine or canine cartilage, an effect that may be due to DEX causing more rapid differentiation of immature chondrocytes and subsequent changes to the ECM. In the same experiment, treated juvenile explants had higher concentrations of orthohydroxyproline (analogous to total collagen concentration) per wet weight after DEX exposure, while adult explants did not.

How the metabolism of healthy cartilage responds to DEX exposure is also unclear. Lu *et al.* (2011) showed no change in the rate of ^35^S-sulphate incorporation at low doses of DEX; only at doses above 100 nM, ^35^S-sulphate incorporation significantly increases as compared to the control. However, Datuin *et al.* (2001) observed the exact opposite effect in a tilapia gill cartilage explant model, where any exposure to DEX, as low as 250 pM, significantly decreases the level of ^35^S-sulphate uptake, suggesting a decrease in metabolic activity and ECM synthesis. Due to the variety of transcriptional changes caused by GCs, it is unsurprising that there would be effects on the rates of protein synthesis within the tissue, even if the direction of the effect reported in the literature is inconsistent, perhaps across different species or types of cartilage used. Siengdee *et al.* (2015) hypothesised that DEX treatment would lead to some level of cell death within the matrix, as confirmed by histological sections revealing clusters of clumped chondrocytes, indicative of cell death. While Datuin *et al.* (2001) did not report effects on cell viability within the tissue, DEX doses above 250 nM significantly decrease the level of ^3^H-thymidine incorporation, associated with decreased cell proliferation.

Cartilage explant models of arthritis show potentially protective effects of DEX on ECM due to the downregulation of MMPs. In addition, the structure of healthy cartilage seems to be maintained under DEX exposure. However, the results on cell behaviour and metabolism within native tissue leave open the possibility that there may be negative effects of the drug on resident chondrocytes, especially at higher doses. Several studies have been performed on chondrocytes isolated from tissue to interrogate the mechanisms of DEX action in both arthritic and healthy contexts, with many conflicting results in different models and DEX doses, as described below.

### DEX and chondrocyte studies of arthritis and inflammation

Human chondrocytes isolated from patients with arthritis or cultured with inflammatory cytokines offer a model system in which the pathways affected by DEX treatment can be examined in more detail (Table 4). However, in keeping with the animal and explant studies, it is difficult to draw specific conclusions because publications often report conflicting results. Stöve *et al.* (2002) isolated primary chondrocytes from the cartilage of OA patients taken at the time of knee joint replacement surgery and cultured them in alginate beads with 0.1 ng/mL IL-1β. They found that the addition of DEX (100 nM–10 μM) lowers the PG content of the culture and decreases the transcription of aggrecan and MMP-3, supporting the hypothesis that DEX does not protect cartilage by increasing the synthesis of ECM components but instead by preventing the synthesis of matrix-degrading factors. Villiger *et al.* (1992), using chondrocytes and cartilage from human donors with no joint disease, showed that culture with IL-1 stimulates increased transcription of biologically active MCP-1, while treatment with DEX decreases MCP-1 synthesis, suggesting the ability to prevent monocyte infiltration and, thus, progression of cartilage degradation. However, certain chondrocyte models reveal that DEX may pose a risk to cell viability. For example, 63 μM DEX was shown to significantly increase the rate of apoptosis in chondrocytes isolated from knee cartilage of OA joint replacement patients by decreasing ERK signalling (Tu *et al.*, 2013). This DEX dose was quite high, but other examples in the literature discussed below show similar effects at doses orders of magnitude lower.

Some studies with human chondrocytes have been performed to elucidate the effect of DEX on inflammatory pathways, although the entire picture remains unclear. 1–100 nM DEX reduces IL-17-induced NO synthesis, inhibiting the production of IL-6 and iNOS transcription, suggesting a potential pathway for the anti-inflammatory effects of DEX in cartilage tissue (Shalom-Barak *et al.*, 1998). 100 nM DEX decreases the transcription of IL-1Ra after IL-6 exposure with human primary chondrocytes, indicating that IL-1Ra production is not a method by which DEX could protect cartilage against damaging inflammatory factors (Palmer *et al.*, 2002). A phosphoproteomics study using primary human chondrocytes treated with IL-1α and 100 nM DEX showed a decrease in the phosphorylation of JNK1 and −2 linked to the anti-catabolic effects of DEX, suggesting a major role for JNKs in regulating cartilage breakdown upon inflammatory challenge [Wang *et al.* (2017b) Phosphoproteomics analysis of signaling changes in human chondrocytes following treatment with Il-1, IGF-1 and dexamethasone. Osteoarthritis Cartilage 25: S165–S166].

Several studies of animal chondrocytes subjected to arthritic-like conditions (Table 4) are consistent with some of the findings from human cells. IL-1α or β-challenged chondrocytes from equine and bovine joint cartilage also show decreased expression levels of MMP-3, −13 and −1 when treated with 100 nM DEX (Busschers *et al.*, 2010; Richardson and Dodge, 2003; Sadowski and Steinmeyer, 2001). However, DEX has similar effects on the family of TIMPs, decreasing their expression levels, further supporting the hypothesis that DEX may cause dysregulation of matrix organisation beyond the scope of MMPs alone. This dose of DEX does not rescue GAG loss in response to IL-1β stimulation of equine chondrocytes (Richardson and Dodge, 2003) but rescues collagen II loss in a model using IL-1α-challenged bovine chondrocytes cultured in agarose gels (Roach *et al.*, 2016). Similar to the results of Glade *et al.* (1983) and Datuin *et al.* (2001), Sadowski and Steinmeyer (2001) found that DEX treatment (100 nM to 50 μM) depresses total protein synthesis by up to 40 % in bovine chondrocytes stimulated with IL-1α, although cell viability is not affected by DEX. Roach *et al.* (2016) also reported that DEX does not change the anti-proliferative effect of IL-1α treatment and that treating the chondrocytes with DEX alone results in the same inhibition of proliferation as inflammatory cytokines.

DEX has also been demonstrated to regulate the transcription of COX2, an inflammation-induced enzyme that produces prostaglandins, in arthritic contexts. 100 nM DEX prevents the transcription of COX2 in primary human chondrocytes after IL-1β exposure, as well as the production of prostaglandin E2, a (mostly) pro-inflammatory agent that has been a palliative target for RA treatment for years (Geng *et al.*, 1995; McCoy *et al.*, 2002; Sheibanie *et al.*, 2007). However, inhibition of COX2 likely does not produce a straightforward anti-inflammatory response, as some prostaglandins produced by COX2 can have anti-inflammatory effects and even prostaglandin E2 is involved in the resolution of inflammation as well as its induction in an RA mouse model (Chan and Moore, 2010).

### Non-arthritic chondrocyte cultures: effects on viability and proliferation

Most studies with human chondrocytes show an apoptotic effect of DEX on isolated cells in a non-arthritic context (Liu *et al.*, 2014; Shen *et al.*, 2015; Zaman *et al.*, 2014), which Liu *et al.* (2014) attributed to the induction of autophagy by DEX (Table 5). In their model, a downstream effect of DEX is to increase the production of ROS, which trigger an autophagic response, leading to cell death. However, Shen *et al.* (2015) proposed that oxidative stress due to increased ROS production is the cause of chondrocyte death and that autophagy serves to protect cells from this stress. This discrepancy could be due to the difference in DEX concentration used, which was two orders of magnitude higher in Liu *et al.* (2014) than Shen *et al.* (2015), but the complex interplays between these pathways, highlighted in Fig. 1, remain to be clarified.

In contrast to these studies, Dragoo *et al.* (2012) did not find any change in the amount of cell death, even at the incredibly high dose of 1.5 mM DEX. Stueber *et al.* (2014) reported no change in cell viability as well, although their experiment only tested the effects of the drug for 1 h, while Song *et al.* (2012) reported viability changes only after 72 h of treatment. However, Dragoo *et al.* (2012) reported their results after 1 week of treatment, so the discrepancy with that experiment remains.

Mushtaq *et al.* (2002) were the only group using isolated animal chondrocytes that reported the effects of DEX on apoptosis and found no change after 20 d of DEX treatment. It is of note that these cells were a chondrogenic teratocarcinoma cell line instead of primary chondrocytes, which may have influenced their behaviour. However, all three groups that have used animal chondrocytes reported a reduction in the rate of cell proliferation after DEX exposure, suggesting that even if DEX does not kill the cells, it makes them more quiescent (Hainque *et al.*, 1987; Maor and Silbermann, 1986; Miyazaki *et al.*, 2000). It is unclear why so many chondrocyte models report an apoptotic effect that is not reflected in tissue explant cultures. This may be due to a lower effective concentration in explants, as the drug must diffuse through the dense cartilage depending on treatment duration or a different homeostatic state of the cells in whole tissue *versus* cell monolayer. Chondrocytes in monolayer culture experience an entirely different set of external stimuli as compared to those suspended in a native 3D pericellular matrix, both through biological and mechanical signalling (Guilak *et al.,* 2006). This could lead to a lack of external survival signals received by the monolayer chondrocytes after DEX exposure, rendering them unable to prevent apoptosis.

The relationship between DEX, autophagy and cell death is further complicated by a recent study on the effects of DEX on senescence in rat knee chondrocytes. Xue *et al.* (2016) found that a range of 0.25–128 μM DEX activates autophagy and senescence in chondrocytes and that senescence increases after inhibiting autophagy with an mTOR inhibitor, potentially demonstrating that DEX-induced autophagy serves to protect chondrocytes from senescence. Senescence has been identified as a correlative factor for OA progression, so understanding the relationship between DEX dose and its activation of autophagy, apoptosis and senescence will be critical to understanding how to safely use it in patients.

### Non-arthritic chondrocyte cultures: effects on ECM gene expression

Changes in the synthesis of ECM components at the cellular level alone do not seem to explain the effect of DEX on cartilage as a whole. Collagen II synthesis either decreases or does not significantly change after DEX treatment (Table 6) (James *et al.*, 2007; Richardson and Dodge, 2003; Song *et al.*, 2012). Song *et al.* (2012) found that DEX decreases the synthesis of aggrecan in healthy human chondrocytes. James *et al.* (2007) used an RNA microarray to assay the entire transcriptome of chondrocytes after DEX treatment and found that ECM genes as a whole are highly enriched in the DEX-treated group, although aggrecan and collagen II are not in the list of the most enriched ECM genes, which includes many of the other collagen family members, fibronectin, matrilin and laminin.

In healthy cartilage explant models, it is unclear whether DEX increases, decreases or does not affect cartilage GAG content, while the results from isolated cells suggest a complicated interplay in the regulation of ECM-remodelling factors by DEX. MMP-13, −1 and −3 are all significantly downregulated after DEX exposure (Richardson and Dodge, 2003) but the results of James *et al.* (2007) showed that several members of the ADAMTS family are among the most highly enriched ECM genes. Adding to the discussion of the dysregulation of matrix organisation by DEX, Hinek *et al.* (1984) reported that 100 nM DEX causes rabbit chondrocytes to deposit more elastic fibres in a more disorganised network. The relationship of the effects of DEX on aggrecan and metalloproteinase expression and the resulting effects on cartilage tissue structure as a whole have not yet been elucidated but is integral to understanding the results of studies such as the disperse collagen networks of Jaffré *et al.* (2003), as well as the safety of DEX for long-term joint treatment.

### Non-arthritic chondrocyte cultures: other affected pathways

Several other pathways relevant to normal cartilage function are affected by DEX in isolated experiments. The results of the transcriptome screen done by James *et al.* (2007) identified the “metabolism” gene set as the most highly enriched in DEX-treated cells as compared to control, although no single metabolic pathway stood out as being upregulated as a whole. Glutathione S-transferases and aldehyde dehydrogenases, involved in several metabolic pathways, were some of the most notable individual genes. Studies using a non-arthritic embryonic mouse chondrocyte model found that SOX9, a transcription factor that activates chondrocyte differentiation, is upregulated after 100 nM DEX treatment for 48 h (Jo *et al.*, 2014; Sekiya *et al.*, 2001). However, Song *et al.* (2012), using adult human chondrocytes, observed the opposite effect, which suggests that the effects of DEX could be dependent on the developmental state of the cells. Similarly, differences were noted by Bian *et al.* (2010) when using juvenile *versus* adult bovine tissue, different types of cartilage or samples from different species.

Fahey *et al.* (2009) reported an increase in CPPD crystal deposition in porcine chondrocytes caused by 96 h treatment with 10 nM to 1 μM DEX as a result of the upregulation of transglutaminase activity. CPPD crystals are suspected to contribute to joint damage in OA; thus, while DEX may help preserve PG and collagen structure of arthritic cartilage, it may also initiate other cell-mediated processes that become detrimental to joints (Fahey *et al.*, 2009).

## Discussion

The results of studies using DEX both *in vivo* and *in vitro* are highly dependent on model system, dosage and duration of DEX exposure. Understanding appropriate doses for glucocorticoids, in general, is vital for performing and comparing the results of clinical trials. For example, two separate trials studied the symptomatic benefits of TA on patients with knee OA (Kellgren-Lawrence grades 2 or 3): while Conaghan *et al.* (2018) found beneficial effects at 12 weeks using a single 32 mg dose of i.a. extended-release TA, McAlindon *et al.* (2017) used 40 mg i.a. injections of TA every 3 months for 2 years and reported a significant increase in cartilage volume loss. It is difficult to assess whether the differences in these outcomes are due to total dose, duration of treatment or both. Hence, a search into the literature was performed for clues from *in vitro* and animal studies. In this regard, the present review focuses on DEX alone, as studies have shown that different GCs with very different potencies can have different effects on cartilage cells and native tissues, as well as in various animal models (Dragoo *et al.*, 2012; Mushtaq and Ahmed, 2002; Sadowski and Steinmeyer, 2001; Siengdee *et al.*, 2009). Thus, it would be difficult to draw definite conclusions from such disparate studies about the safety or efficacy of the GC family as a whole.

Biological processes in cartilage that have been identified as being affected by DEX are highlighted in Fig. 1. While the results of studies on ECM synthesis in healthy isolated chondrocytes are inconsistent (James *et al.*, 2007; Richardson and Dodge, 2003; Song *et al.*, 2012), chronic administration of DEX at high doses was shown in some *in vivo* studies to have catabolic effects on initially healthy tissue (Annefeld and Erne, 1987; Glade *et al.*, 1983; Podbielski *et al.*, 1985). However, DEX may offer protection against the degenerative effects of arthritic diseases on ECM structure. At the tissue and cell level, DEX inhibits the production of matrix-degrading factors, rescuing matrix breakdown in arthritic contexts [Li *et al.*, 2015; Lu *et al.*, 2011; Wang *et al*. (2014) Dexamethasone treatment alters the response of human cartilage explants to inflammatory cytokines and mechanical injury as revealed by discovery proteomics. Osteoarthritis Cartilage 25: S381–S382]. While ECM content may be maintained, there may still be disruption of the matrix structure itself due to the dysregulation of cell-mediated remodelling factors, the effects of which on cartilage tissue in the long-term remain to be studied (Jaffré *et al.*, 2003). One of the greatest challenges in comparing the results of various studies is the variety of model systems being used: isolated chondrocytes *versus* cartilage explant organ culture or co-culture of different joint tissues *versus* animal models and human clinical trials. While chondrocyte monoculture is useful for easier interrogation of molecular pathways, this approach cannot recapitulate the complex interactions of cells within their native 3D tissue matrix or between the different tissues of the joint. Even cultures incorporating chondrocytes suspended in a hydrogel to recapitulate the earliest stages of a 3D neo-tissue environment do not capture the same processes as it occurs in native tissues due to the differences in ECM composition, which complicates conclusions drawn about ECM synthesis and remodelling (Buschmann *et al.*, 1995).

The overall effects of DEX on cell processes and viability remain unclear, as there are reports of both high and low doses of DEX either reducing or maintaining cell viability. DEX protects cartilage viability in an IL-1α challenge of bovine cartilage explants (Li *et al.*, 2015), but many studies using isolated chondrocytes have reported significant losses of cell viability after DEX exposure (Liu *et al.*, 2014; Shen *et al.*, 2015; Song *et al.*, 2012; Zaman *et al.*, 2014). One mechanism that may underlie loss of cell viability is the dysregulation of metabolism triggered by DEX, leading to upregulation of ROS within the chondrocytes. This process can trigger an autophagic response and, currently, conflicting reports exist in the literature on whether autophagy is the cause of chondrocyte apoptosis after DEX exposure or is protective against ROS-induced apoptosis and the answer likely depends on the dose of DEX used (Liu *et al.*, 2014). ROS generation has been linked to catabolic effects in OA (Lepetsos and Papavassiliou, 2016), so the dose-dependent balance of ROS and autophagy in whole cartilage under arthritic conditions must be better understood. This, as well as the increase in CPPD crystal formation after DEX treatment reported by Fahey *et al.* (2009), could worsen the progression of the disease and more work must be done with human tissue to determine the extent of the risk. Autophagy is suppressed in arthritic contexts (Cheng *et al.*, 2017), so the pro-autophagic response of DEX may serve to restore healthy homeostatic mechanisms and protect cartilage tissue, although this possibility has not yet been explored in the literature.

Studies with isolated cells seem to agree that DEX can reduce proliferation, which is also seen with higher DEX doses in cartilage tissue (Datuin *et al.*, 2001). The anti-proliferative effect of DEX would be of great concern when treating young patients, as cartilage development could be disturbed upon DEX exposure (Datuin *et al.*, 2001; Hainque *et al.*, 1987; Maor and Silbermann, 1986; Miyazaki *et al.*, 2000). Importantly, it is not known under what conditions and doses DEX drives cells towards quiescence, senescence or apoptosis. *In vitro* and *in vivo*, DEX exposure activates the p53/p21 pathway in tenocytes, a driving pathway towards senescence, although the effects of DEX on this pathway in cartilage are unknown (Poulsen *et al.*, 2014). If DEX-induced reduction in proliferation as measured by ^3^H-thymidine incorporation is a result of a shift towards senescence, it could seriously affect the ability of cartilage tissue to respond to future challenges.

One aspect of DEX treatment that has not been well-explored in the literature is the duration of specific cell and tissue responses after a given exposure to DEX. Some studies have been performed with other GCs, such as that of Behrens *et al.* (1976), using rabbits injected every week with 25 mg hydrocortisone for 9 weeks. 26 weeks after hydrocortisone injections were stopped, metabolic rates increased towards normal along with an increase in cell proliferation, which was attributed to cells replicating to replace those killed by the multiple high-dose steroid injections, and the remaining cells restoring a more normal biosynthetic capacity. However, the extent to which the disrupted remodelling of the ECM can be rescued after such aggressive steroid treatment has not yet been explored, so it is unknown how the effects of the disruption of tissue remodelling factors may last and whether this could render the tissue more prone to disease in the future.

A question that must be discussed when studying any aspect of arthritis *in vitro* is whether the results would be different in a model of the entire joint, taking into account the cross-talk between cartilage, joint capsule synovium, bone and immune cells (Pelletier, 1999). The disease-modifying potential of DEX does not depend only on its effects on cartilage, but on the state of the joint as a whole. DEX was shown to reduce the transcription of some inflammatory cytokines in cartilage explants under arthritic stress, which would protect not only cartilage viability but potentially reduce inflammatory reactions of the surrounding joint tissues (Lu *et al.*, 2011). As mentioned above, DEX reduces the production of MCP-1 in chondrocytes, reducing the potential for macrophage infiltration in RA, a possible mechanism of reducing RA disease progression (Koch *et al.*, 1992). DEX also reduces cytokine expression by macrophages in an inflammatory environment, providing a potential protective mechanism against cytokine-induced damage in RA (Bartneck *et al.*, 2014). The ROS-producing effect of DEX, while linked to apoptosis in chondrocytes, serves to increase the T-cell suppressive capacity of anti-inflammatory macrophages, which has been linked to reducing the severity of RA in a mouse model (Gelderman *et al.*, 2007; Kraaij *et al.*, 2011). The synovium also secretes a large quantity of inflammatory cytokines immediately following a traumatic joint injury, a process which DEX suppresses in a rabbit model of post-traumatic OA, contributing to its apparent chondroprotective action (Huebner *et al.*, 2014). *In vitro*, human cartilage-bone-synovium co-culture models of PTOA have also highlighted the potential of DEX in reducing cytokine release by synovium explants and thereby inhibiting proteolytic aggrecanGAG loss of cartilage [Dwivedi *et al.* (2019) Human cartilage-bone-synovium microphysiological system to study PTOA pathogenesis and treatment on Earth and in space. OAC 27: S167]. DEX also inhibits the production of COX-2 in the synovium in response to IL-1β, a non-disease-modifying process that is used in several drugs on the market for treating OA and RA symptoms (Crofford *et al.*, 1994; Laine *et al.*, 2008; Sundy, 2001).

One of the most serious side effects of chronic GC treatment in humans is bone loss, which also occurs during arthritis progression (American College of Rheumatology *Ad Hoc* Committee on Glucocorticoid-Induced Osteoporosis, 2001; Hoes *et al.*, 2015; Oelzner *et al.*, 2010). After DEX treatment, healthy bone metabolism is affected, leading to changes in remodelling and eventual bone density loss and a reduction in load potential (Liu *et al.*, 2015). It is unclear whether DEX worsens the progression of osteoporosis in arthritic animal models, but it is nonetheless worth exercising caution when considering the balance of side effects *versus* potential therapeutic gains (Islander *et al.*, 2011; Oelzner *et al.*, 2010; Quan *et al.*, 2016). DEX also has been shown to stunt growth of developing bone and cartilage, explaining the effects of growth retardation and osteopenia seen in children treated with extended doses (Mushtaq and Ahmed, 2002; Tomaszewska *et al.*, 2013). Side effects of DEX may also be ameliorated by combination treatments with other drugs such as antioxidants that combat its effects on metabolism, a combination that has already shown, *in vivo*, to improve the therapeutic capacity of DEX alone (Roy *et al.*, 2013).

Finally, given the need for low dose treatment without use of multiple i.a. injections, improved drug delivery techniques are critically important for therapeutic treatment of OA/PTOA. Developing tools, such as nanocarriers or functionalised protein carriers, to aid in delivery of DEX specifically to the cartilage tissue will allow the drug to be given in single injection low-dose modalities, which may prevent its negative effects on cartilage tissue as well as systemic side effects (Bajpayee *et al.*, 2016; Bajpayee and Grodzinsky, 2017; Geiger *et al.*, 2018; Krishnan *et al.*, 2018).

## Conclusions

Considering the literature on DEX and the history of DEX use in OA and RA, treatment plans for clinical applications emphasise that beneficial effects of GCs are supported for i.a. administration at the lowest efficacious dose (Wernecke *et al.*, 2015). Regarding OA, it is important to distinguish between two different patient populations: those with well-established OA, mainly seeking pain relief, and those who have suffered a traumatic joint injury in a previously healthy joint. For the latter population, clinical and *in vitro* studies are looking toward the possibility that DEX (or other GCs) may ultimately prove to be disease-modifying if used immediately after injury and at low dose for a short enough duration (Grodzinsky *et al.*, 2017; Lattermann *et al.*, 2017). Thus, after a traumatic joint injury, which can progress to PTOA, treatment with DEX may prevent initial damage done by the release of inflammatory cytokines and maintain cartilage structure, lowering the chance of developing PTOA (Li *et al.*, 2015). However, due to the potential for catabolic effects on cartilage and bone along with systemic side effects, chronic repeated high-dose administrations of DEX may do more harm than good to the joint and its use in the treatment of children is not advised due to its effects on growth and development. More work must be done in human models and clinical trials to discover what the long-term effects of DEX-induced dysregulation of cellular metabolism and ECM remodelling may be and whether DEX can aid in preventing PTOA after a traumatic joint injury. After decades of use in OA and RA patients, full understanding of the therapeutic potential of DEX, along with other GC family members, may only now be coming to light, although much more careful and consistent study and optimisation of treatment remain to be done.

## Figures and Tables

**Table 1.** DEX studies in animal models summarising model used, DEX dosage and duration, frequency and administration route of DEX treatment and overall observed effect.

Authors	Year	Animal model	Arthritis model	DEX dose	Duration	Frequency and route of administration	Observed effect
Huebner *et al*.	2014	Rabbit	Yes	0.5 mg/kg	3 weeks	Once/3 d, i.a.	+
Heard *et al*.	2015	Rabbit	Yes	0.5 mg/kg	48 h-9 weeks	Once, i.a.	+/−
Islander *et al*.	2010	Mouse	Yes	~ 5 mg/kg	~ 25 d	Daily, i.p.	+
Rauchhaus *et al*.	2009	Mouse	Yes	0.4–4 mg/kg	7 d	Once, i.v.	+/−
Wang *et al*.	2017a	Rat	Yes	0.15 mg/kg	7 d	Daily, oral gavage	+
Malfait *et al*.	2009	Rat	Yes	1 mg/kg or 0.001–1 mg/mL	16 h	Once, oral gavage or i.v. infusion	+
Ashraf *et al*.	2011	Rat	Yes	0.1 mg/kg	24 d	Daily, oral gavage	+
Jaffré *et al*.	2003	Rat	Yes	0.1 mg/kg	5–21 d	Daily, i.a.	+/−
Annefeld and Erne	1987	Rat	No	3 mg/kg	3 weeks	1/week, i.m.	−
Podbielski and Raiss	1985	Rat	No	3.3 mg/kg	3–5 weeks	1/week, i.m.	−
Glade *et al*.	1983	Horse	No	0.005 mg/kg	3–11 months	Daily, i.m.	−

i.a.: intra-articular injection. i.p.: intra-peritoneal injection. i.v.: intravenous injection. i.m.: intra-muscular injection. + indicates a positive effect on cartilage and/or arthritis progression, − a negative effect and +/− some positive results alongside negative side effects.

**Table 2.** DEX studies using explants from human or animal cartilage in models of arthritis. Summary of DEX concentration, duration of culture, inflammatory cytokines used to model arthritic conditions and observed effect on measurements associated with arthritis progression. APC: activated protein C. + indicates a rescue of arthritis-induced effects, +/= rescue of only some effects and +/− rescue of some effects and worsening of others. Wang *et al.* (2014) Dexamethasone treatment alters the response of human cartilage explants to inflammatory cytokines and mechanical injury as revealed by discovery proteomics. Osteoarthritis Cartilage 25: S381–S382.

Authors	Year	Tissue type	Cytokine treatment	DEX dose	Duration	Observed effect
Li *et al.*	2015	Human	ng/mL IL-1α	nM	d	+
		Bovine	ng/mL IL-1α	nM	d	+/=
Wang *et al.*	2014	Human	ng/mL TNFα + 50 ng/mL IL-6	nM	d	+
Lu *et al.*	2011	Human	ng/mL TNFα ± 50 ng/mL IL-6	nM	d	+
		Bovine	ng/mL TNFα ± 50 ng/mL IL-6	nM	d	+
Busschers *et al.*	2010	Equine	ng/mL IL-1β	nM-1 μM	h	+/=
Garvican *et al.*	2010	Equine	ng/mL IL-1 + 10 μg/mL APC	1–100 μM	d	+/−
Saito *et al.*	1999	Rabbit	ng/mL IL-1α + 100 μg/mL plasminogen	0.1–100 nM	d	+

**Table 3.** DEX studies using cartilage explants in non-arthritic model systems. Summary of DEX concentration, duration of culture and overall observed effect on cartilage. = indicates no change in measured outcomes, +/= some healthy effects and some outcomes that do not change and − adverse effects on the cartilage tissue.

Authors	Year	Tissue type	DEX dose	Duration	Observed effect
Siengdee *et al.*	2015	Porcine	1.25–5 mM	2 weeks	−
Lu *et al.*	2011	Bovine	10 nM	6 d	=
Bian *et al.*	2010	Bovine	0.1 μM	4 weeks	+/=
Busschers *et al.*	2010	Equine	100 nM-1 μM	72 h	−
Datuin *et al.*	2001	Tilapia	0.25 nM–2.5 μM	Not reported	−

**Table 4.** DEX studies using chondrocytes isolated from arthritic patients and/or cultured with inflammatory cytokines. Summary of cell source (OA: from patient with symptomatic OA), inflammatory cytokines used to model arthritic conditions, DEX concentration and duration of culture. N/A: not available. Wang *et al.* (2017b) Phosphoproteomics analysis of signaling changes in human chondrocytes following treatment with Il-1, IGF-1 and dexamethasone. Osteoarthritis Cartilage 25: S165–S166.

Authors	Year	Cell type	Cytokine treatment	DEX dose	Duration
Wang *et al.*	2017b	Human	10 ng/mL IL-1α	100 nM	30 min
Tu *et al.*	2013	Human, OA	N/A	63 μM	48 h
Stöve *et al.*	2002	Human, OA	0.1 ng/mL IL-1β	100 nM-10 μM	1 week
Palmer *et al.*	2002	Human, OA	10 ng/mL IL-1β or 100 ng/mL IL-6	100 nM	18 h
Shalom-Barak *et al.*	1998	Human	10 ng/mL IL-17	1–100 nM	48 h
Villiger *et al.*	1992	Human	10 ng/mL IL-1	100 nM	5 h
Roach *et al.*	2016	Bovine	10 ng/mL IL-1α	100 nM	35 d
Busschers *et al.*	2010	Equine	5 ng/mL IL-1β	100 nM-1 μM	24 h
Richardson and Dodge	2003	Horse	10 ng/mL IL-1β or 25 ng/mL TNFα	10 nM-1 μM	24 h
Sadowski and Steinmeyer	2001	Bovine	0.5 ng/mL IL-1α	100 nM–50 μM	48 h

**Table 5.** Results on cell viability and proliferation after DEX treatment on primary chondrocytes in non-arthritic model systems. − denotes a decrease and = no change from control.

**Authors**	**Year**	**Cell type**	**DEX dose**	**Duration**	**Effect on viability**
Shen *et al.*	2015	Human	1 μM, 10 μM	24 h	−
Liu *et al.*	2014	Human	100 μM	72 h	−
Stueber *et al.*	2014	Human	10 μM–1 mM	1 h	=
Zaman *et al.*	2014	Human	1 μM	24–72 h	−
Song *et al.*	2012	Human	20–200 μM	24 h-1 week	−
Dragoo *et al.*	2012	Human	1.5 mM	1 week	=
**Authors**	**Year**	**Cell type**	**DEX dose**	**Duration**	**Effect on proliferation**
Mushtaq *et al.*	2002	Mouse	10 nM–1 μM	20 d	−
Miyazaki *et al.*	2000	Rat	0.1 nM–100 μM	36 h	−
Hainque *et al.*	1987	Rabbit	10 nM–100 μM	24 h-2 weeks	−
Maor and Silbermann	1986	Mouse	1 μM	24 h	−

**Table 6.** ECM-related gene expression of primary chondrocytes after DEX exposure. + denotes an increase, − a decrease and = no change from control. James *et al.* (2007) used an ECM gene set from the Gene Set Enrichment Analysis (GSEA) database (Subramanian *et al.*, 2005).

Authors	Year	Cell type	DEX dose	Duration	Effect on RNA expression
Song *et al.*	2012	Human	20–200 μM	24 h-1 week	− ACAN, − collagen II
James *et al.*	2007	Mouse	100 nM	24 h	+ ECM genes
Richardson and Dodge	2003	Equine	10 nM–1 μM	24 h	− collagen II, − MMP13, − MMP1, − MMP3, + fibronectin

## List of Abbreviations

AA: adjuvant-induced arthritis
ADAMTS-5: a disintegrin and metalloproteinase with thrombospondin motifs 5
APC: activated protein C
COX2: cyclooxygenase II
CPPD: calcium pyrophosphate dihydrate
DEX: dexamethasone
DMOAD: disease-modifying drug
ECM: extracellular matrix
ERK: extracellular signal-regulated kinases
GAG: glycosaminoglycan
GCs: glucocorticoids
i.a.: intra-articular
IL: interleukin
IL-1Ra: IL-1 receptor antagonist
iNOS: inducible nitric oxide synthases
JNK: c-Jun N-terminal kinase
LDH: lactate dehydrogenase
MMP: matrix metallopeptidase
MCP-1: monocyte chemoattractant protein-1
MRI: magnetic resonance imaging
NF-κB: nuclear factor kappa-light-chain-enhancer of activated B cells
NO: nitric oxide
OA: osteoarthritis
PG: proteoglycan
PTOA: post-traumatic osteoarthritis
RA: rheumatoid arthritis
ROS: reactive oxygen species
sGAG: sulphatedGAG
TA: triamcinolone
TIMPs: tissue inhibitors of metalloproteinases
TNFα: tumour necrosis factor alpha
